# Regulatory roles of 24-epibrassinolide in tolerance of *Acacia gerrardii* Benth to salt stress

**DOI:** 10.1080/21655979.2017.1297348

**Published:** 2017-11-01

**Authors:** Elsayed Fathi Abd_Allah, A. A. Alqarawi, Abeer Hashem, Stephan Wirth, Dilfuza Egamberdieva

**Affiliations:** a Plant Production Department, Faculty of Food and Agriculture Sciences, King Saud University, Riyadh, Saudi Arabia; b Seed Pathology Department, Plant Pathology Research Institute, ARC, Giza, Egypt; c Botany and Microbiology Department, Faculty of Science, King Saud University, Riyadh, Saudi Arabia; d Mycology & Plant Disease Survey Department, Plant Pathology Research Institute, ARC, Giza, Egypt; e Institute of Landscape Biogeochemistry, Leibniz Centre for Agricultural Landscape Research (ZALF), Müncheberg, Germany

**Keywords:** *A. gerrardii*, Antioxidant enzymes, oxidative stress, membrane stability index, ROS, salinity

## Abstract

This experiment aimed to investigate the role of 24-epibrassinolide (EBL) against NaCl^−^induced salinity stress in *Acacia gerrardii* Benth. NaCl (200 mM) imparted deleterious effects on the growth and chlorophyll contents of *A. gerrardii*, but foliar application of EBL (1.0 mg/l; each plant received 2.5 ml) mitigated the negative effect considerably. NaCl reduced chlorophyll content but this was significantly ameliorated by the application of EBL. EBL reduced significantly NaCl^−^induced oxidative stress hence protect membranes and also improved the relative water content significantly by 6.6% as compared with control. Nitrate reductase activity declined after NaCl treatment but EBL application sustained its activity under normal and stressed conditions. Exogenous application of EBL significantly improved the activity of superoxide dismutase, catalase and the enzymes of the ascorbate-glutathione pathway thereby protecting the photosynthetic electron transport chain and other metabolic processes in *A. gerrardii* from NaCl^−^induced oxidative stress.

## Background

Salinity stress is a major abiotic limiting factors for sustainable crop productivity. Salt stress affects plant growth by inducing osmotic and ionic stress leading to an ion imbalance and oxidative stress.^1^ High soil salinity alters various morphological, physiologic and biochemical processes including germination, photosynthesis, redox homeostasis, mineral uptake, lipid metabolism, and the accumulation of low molecular mass compounds like proline and glycine βine, antioxidants, and protein.^1,2^ Besides affecting plant growth, high salt concentration hampers soil health by affecting soil porosity and water potential, resulting in changes at the cellular and whole plant level and hence hampering the physiologic status of the plant.^3^ Once taken up by plants, the excess accumulation of sodium ions inhibits the activity of enzymes involved in nitrogen metabolism and photosynthetic carbon fixation.^4,5^ In addition, excess sodium prevents the uptake of essential elements such as potassium and calcium by direct competition of transporters at the plasma membrane, leading to an ionic imbalance.^6,7^ Salt stress-induced ionic imbalances lead to hyperosmotic stress that stimulates the production of reactive oxygen species (ROS), alters redox homeostasis, induces dehydration, and causes a loss of turgor.^8^ Under high salt concentrations, ionic imbalances lead to cell death by causing a decline in the Na^+^/K^+^ ratio, inactivating enzyme activity and affecting metabolic processes of plants.^1,8^

Among the major agents causing salinity, NaCl has been considered as a major contributor to this abiotic threat. It has been proposed that plants avoid the negative effects of salinity either by enhancing the partitioning and compartmentation of salts to less sensitive tissues and cellular spaces or by directly avoiding their uptake.^9,10,11^ Also, excess accumulation of NaCl is linked with the manifold increase in ROS hence affecting the normal physiologic functioning of plants by affecting enzyme activity, membrane functioning and DNA.^12^ The production of ROS, like superoxide (O_2_^−^), hydrogen peroxide (H_2_O_2_), and hydroxyl (HO^−^), increases considerably due to excess NaCl uptake.^11,13^ Likewise, the mechanisms exist within the system to eliminate ROS and avert cellular oxidative damage. In response to high salinity, like other stresses, such ROS-scavenging and tolerance mechanisms are upregulated in plants for attenuating the deleterious effects of salts and the constituents of such intriguing mechanisms include antioxidants and osmolytes.^14,15^ Antioxidant enzymes, including superoxide dismutase (SOD), catalase (CAT), peroxidases (PODs) and reductases, which include glutathione reductase (GR), monodehydroascorbate reductase (MDHAR) and dehydroascorbate reductase (DHAR), mediate the elimination of ROS and mitigate the negative effects of NaCl on plant cellular functioning.^16^ Hashem et al.^7^ noted that upregulation of antioxidants rapidly neutralizes ROS, hence averting salinity-induced oxidative damage. Several reports have suggested the beneficial role of improved antioxidant metabolism, together with the accumulation of osmotica, in bringing stability to major physiologic functions like respiration, photosynthesis and membrane functioning in plants.^1,7,17^

Brassinosteroids (BRs) are polyhydroxylated steroidal lactones that exhibit a ubiquitous distribution in plants. BRs are implicated in the regulation of several growth-related and developmental processes in plants including germination, cell elongation, tissue differentiation and morphogenesis, leaf development, senescence, vasculature and male sterility.^18^ In addition to this, BRs have been reported to protect plants against biotic and abiotic stresses.^2,19^ The exogenous application of BRs can modulate the genetic and molecular aspects of metabolic processes through regulated signal crosstalk with other important phytohormones.^20^ Hayat et al.^19^ demonstrated that the application of BRs improves photosynthesis by improving chlorophyll content, CO_2_ assimilation and carboxylation efficiency by enhancing the accumulation of proline and up-regulating the antioxidant defense system resulting in enhanced yield.^19^ Exogenous application of 24-epibrassinolide (EBL) protected *Cucumis sativus* from salinity-induced oxidative damage by enhancing the antioxidant system and resulting in improved membrane stability.^21^ The role of BRs in improving the growth and yield of plants has been attributed to its significant promotive effects on metabolic aspects, including the uptake and assimilation of essential nutrients, and modulation of the enzymatic and non-enzymatic components of antioxidants, osmotic and photosynthetic regulation.^19,21,22,23^ BRs improve mineral uptake by enhancing the activity of transport proteins such as H^+^-ATPase and Ca^2+^-ATPase.^2,18,19,21^

*Acacia gerrardii* Benth, a small tree belonging to the Fabaceae, is commonly found in tropical and subtropical regions of the globe. The present investigation was performed to analyze the impact of salt stress on growth, pigment content and the antioxidant metabolism and the role of exogenously applied EBL on mitigating this stress.

## Material and methods

### Chemical, soil, plant and growth conditions

EBR (C_28_H_48_O_6_, molecular weight = 480; Sigma Chemical Co., state, USA) was initially dissolved in ethanol (95%, v/v) and made up with distilled water containing polyoxyethylenesorbitan monolaurate [Tween 20, Sigma Chemical Co.] at 0.02% (v/v) as adhesive agent. The soil used in this study was a loamy sand type collected from the native habitat of *A. gerrardii* in the Riyadh area, Riyadh, Saudi Arabia, and had the following properties (%): sand (86.2); clay (8.9); silt (4.9); organic carbon (0.14); total nitrogen (0.008); pH 7.6. The seeds of *A. gerrardii* were collected from native trees grown in Rawdhat Khuraim (Riyadh region), Saudi Arabia. The surface of seeds was sterilized with concentrated H_2_SO_4_ followed by 70% (v/v) ethyl alcohol for 3 min, then rinsed many times with sterile H_2_O. Surface-sterilized seeds were germinated on Blotter filter paper (9.0 cm in diameter and 1.4 mm thickness) for 10 d at 25 ± 2°C in a growth incubator. Uniformly germinated seeds were sown in acid-washed sterile sand and kept in a plant growth chamber for one month at 25 ± 2°C and 70–75% relative humidity under an 18 h photoperiod, at 750 μmolm^−2^ s^−1^ photosynthetic photon flux density. Hoagland's solution was used to irrigate potted plants. Seedlings were transplanted to plastic pots (2 Kg capacity, one seedling/pot) and kept in a plant growth chamber for an additional 15 weeks. NaCl was added to Hoagland solution to obtain a concentration of 200 mM. This solution was used to irrigate potted plants. EBR was applied by spraying the shoot system in the early morning at 1.0 mg/l, and each plant received 2.5 ml. Plants were sprayed once a week until plants were harvested. Ten pots were used for each treatment, and each treatment had its own control. At end of the experiment,(nearly 20 weeks) plants were removed very carefully from pots, and morphological criteria were assessed and recorded. Fresh samples of shoots and roots were dried at 110°C until to 2 constant successive weights to determine dry weight.

### Estimation of stomatal conductance and transpiration rate

The third leaf from the top of each seedling was used to estimate stomatal conductance (gs) and transpiration rate (E), using an open system LCA-4 ADC portable infrared gas analyzer (Analytical Development Co., Hoddesdon, UK) as described by Tzortzakis.^6^

### Estimation of photosynthetic pigments

Analysis of photosynthetic pigments in fresh leaves of *A. gerrardii* was done following the method of Lichtenthaler and Wellburn.^24^ Briefly, pigments were extracted in acetone and the absorbance was read spectrophotometrically at 622, 664, and 440 nm. Following formulae were used for calculating the concentrations of chlorophylls and carotenoids.

Chl a (mg/g f.wt.) = [12.7(OD 664)−2.69(OD 622) × V/ 1000 x W]

Chl b (mg/g f.wt.) = [22. 9(OD 622)−4.68(OD 664) × V/ 1000 × W]

Carotenoids (mg/g f.wt.) = Acar/Em × 100

Where, V represents volume of the aliquot and W, weight of tissue

Acar = OD 440 + 0.114(OD 664)−0.638(OD622) and Em = 2500

Determination of leaf relative water content (RWC)

RWC was estimated in fresh leaves in 10 replicates according to the method of Smart and Bingham,^25^ and calculated using the following formula:

RWC (%)= [(fresh weight−dry weight)/ (turgid weight−dry weight)] × 100.

Determination of membrane stability index (MSI)

MSI was estimated by the method described by Sairam et al.^26^ and calculated using the following formula:

MSI = [1 - (C_1_/C_2_)] × 100.

where C_1_ = electrical conductivity of water containing the treatment sample, and C_2_ = electrical conductivity of water containing the blank sample.

### Determination of antioxidative enzyme activities

At the harvest, the leaves were collected and frozen until use for extraction and estimation of antioxidative enzyme activities. Frozen leaf tissue (0.4 g) was homogenized in 4 ml of chilled extraction buffer (50 mM potassium phosphate, pH 7.0, 4% (w/v) polyvinylpyrrolidone (PVP, C_6_H_9_NO, Sigma-Aldrich) using a pre-chilled mortar and pestle. The homogenate was centrifuged for 30 min at 14,000× g at 4°C. The supernatant was used as the enzyme source.

SOD (EC1.15.1.1) activity was assayed by measuring the ability of enzyme extract to inhibit the photochemical reduction of nitroblue tetrazolium (NBT) following the method of Beauchamp and Fridovich.^27^ The reaction mixture contained 50 mM phosphate buffer (pH 7.4), 13 mM methionine, 75 µM NBT, 0.1 mM EDTA, 2 µM riboflavin and 100 µL enzyme extract in a final volume of 1 mL. Samples were incubated for 15 min under fluorescent tubes and absorbance was read at 560 nm (T80 UV/VIS spectrometer, PG Instruments Ltd., USA, used). SOD activity was expressed as U mg^−^1 protein.

CT (EC1.11.1.6) activity was assayed by monitoring the decomposition of H_2_O_2_ at 240 nm. 1 mL of reaction mixture contained 100 mM phosphate buffer (pH 7.0), 0.1 mM EDTA, 0.1% H_2_O_2_ and 100 µL of enzyme extract.^28^ The molar extinction coefficient (ε = 39.4 mM^−1^ cm^−1^) was used for the calculation and expressed as U mg^−1^ protein.

APX (EC1.11.1.1) activity was determined by the method of Nakano and Asada.^29^ The reaction mixture (2 mL) contained 50 mM phosphate buffer (pH 7.8), 0.1 mM EDTA, 0.3 mM ascorbate (AsA), 0.1 mM H_2_O_2_ and 100 µL of enzyme extract and was followed at 290 nm for 3 min. For the calculation of APX activity, the molar extinction coefficient for AsA (ε = 2.8 mM^−1^ cm^−1^) was used, and activity was expressed as U mg^−1^ protein.

GR, (_EC_ 1.6.4.2) activity was assayed according to the method of Smith et al.^30^ by following the increase in absorbance at 412 nm due to the reduction of 5,5′-dithiobis-2-nitrobenzoic acid (DTNB) by reduced glutathione (GSH) generated from the oxidized form of glutathione (GSSG). The reaction mixture consisted of 0.1 M sodium phosphate buffer (pH 7.5), 1 mM EDTA, 0.75 mM DTNB in 0.01 M sodium phosphate buffer (pH 7.5), 0.1 mM NADPH, and 1 mM GSSG. The reaction was started by the addition of enzyme extract. The enzyme activity expressed as Unit/mg protein. One GR activity unit is defined as the amount of enzyme catalyzing the reduction of one micromole of GSSG per minute at pH 7.6 and 25°C.

DHAR, (_EC_ 1.8.5.1) activity was analyzed by following the method of Nakano and Asada.^29^ The change in absorbance was read at 265 nm for 2 min and an extinction coefficient of 14 µM^−1^ cm^−1^ was used for the calculation. The amount of enzyme able to reduce 1 lM DHA per minute (E = 14 µM^−1^cm^−1^) was taken to represent a unit.

To estimate the activity of monodehydroascorbate reductase (MDHAR; EC1.6.5.4), the change in absorbance was recorded at 340 nm.^31^ MDHAR activity was expressed as μmol NADPH oxidized/(EU mg^−1^ protein). One unit of enzyme activity was defined as the amount of enzyme that oxidises 1 nmol of NADH per min at 25°C.

Statistical analysis: All experiments were repeated 3 times and data presented is the mean of 10 replicates. Treatment means were statistically analyzed using Least Significant Difference (LSD) analysis of variance for a completely randomized design by SPSS-22 software.

## Results

Results showing the effect of NaCl (200 mM) and EBL on several growth parameters are depicted in Table 1. Exposure of plants to NaCl reduced shoot and root length by 61.6% and 57.7%, respectively, but the application of EBL reduced this negative effect by improving shoot and root length by 46.9% and 34.3% relative to NaCl^−^treated plants. Under normal conditions (in absence of salt), the observed percent increase was 27.1% for shoot length, 25.3% for root length, 36.7% for shoot dry weight and 36.6% for root dry weight because application of EBL. NaCl reduced the shoot and root dry weight by 67.4% and 69.8%, respectively, but the negative impact wasmitigated by 59.0% and 56.3% after the application of EBL (Table 1).

**Table 1.** Effect of NaCl (200 mM) on morphological criteria (shoot height(SH) [cm], root depth(RD) [cm], shoot dry weight (DW) [g] of and rootDW[g] *A.gerrardii* treated with and without 24-epibrassinolide. Data presented is the mean of 10 replicates.

	Morphological criteria					
	Shoot		Root	SH/RD		
Treatments	Height	DW	Depth	DW	SDW/RDW	
Control	47.1 ± 2.11b	1.6 ± 0.14b	45.7 ± 2.07b	1.26 ± 0.07b	1.03 ± 0.04ab	1.28 ± 0.01b
EBL	64.7 ± 2.64a	2.5 ± 0.18a	61.2 ± 2.81a	1.99 ± 0.12a	1.05 ± 0.04ab	1.27 ± 0.01b
NaCl	18.1 ± 1.72d	0.5 ± 0.07d	19.3 ± 1.42d	0.38 ± 0.02d	0.93 ± 0.03c	1.38 ± 0.02ab
NaCl + EBL	33.8 ± 1.96c	1.2 ± 0.11c	29.4 ± 1.68c	0.87 ± 0.06c	1.15 ± 0.04a	1.46 ± 0.06a
LSD at: 0.05	3.78	0.23	4.82	0.15	0.01	0.01

Plants exposed to NaCl exhibited a 49.1%, 68.6%, 56.0% and 64.9% reduction in chlorophyll a, chlorophyll b, total chlorophylls and carotenoids, respectively but the application of EBL mitigated this deleterious effect by improving the same photosynthetic parameters by 36.8%, 48.6%, 39.5%, and 44.5% more than their respective NaCl^−^treated plants. When EBL was supplied to control plants, chlorophyll a, chlorophyll b, total chlorophylls and carotenoids increased by 17.5%, 22.9%, 39.5% and 45.0%, respectively (Table 2).

**Table 2.** Effect of NaCl (200 mM) on chlorophyll a (Chl a), chlorophyll b (Chl b), carotenoids (Carot) and total chlorophyll content (mg g^−1^ FW) in *A.gerrardii*Benth treated with and without 24-epibrassinolide. Data presented is the mean of 10 replicates.

	Photosynthetic pigments (mg g fresh weight^−1^)					
Treatments	Chl a	Chl b	A+B	A/B	Carot	Total pigments
Control	1.69 ± 0.18b	0.47 ± 0.03b	2.17 ± 0.11b	3.56 ± 0.12	0.19 ± 0.01b	2.36 ± 0.06b
EBL	2.05 ± 0.24a	0.61 ± 0.04a	2.66 ± 0.11a	3.34 ± 0.12	0.29 ± 0.3a	2.95 ± 0.05a
NaCl	0.77 ± 0.08d	0.17 ± 0.02d	0.95 ± 0.05d	4.59 ± 0.27	0.06 ± 0.01d	1.01 ± 0.03d
NaCl + EBL	1.22 ± 0.16c	0.33 ± 0.03c	1.55 ± 0.16c	3.64 ± 0.23	0.12 ± 0.01c	1.67 ± 0.04c
LSD at: 0.05	0.27	0.11	0.35	0.07	0.02	0.42

Exogenous application of EBL resulted in a 6.6% and 8.4% increase in RWC and MSI, respectively. However, NaCl treatment reduced RWC and MSI by 20.3% and 43.1%, respectively relative to the control, while application of EBL reduced the effect of NaCl by improving RWC and MSI 11.9% and 39.0% more than the respective NaCl^−^stressed plants (Table 3). The application of EBL resulted in a 37.9% and 13.8% enhancement of stomatal conductance and transpiration rate relative to the control. These parameters declined 52.2% and 48.6% after exposure to NaCl but were improved by 40.7% and 39.7%, respectively after the application of EBL to NaCl^−^stressed plants (Table 3).

**Table 3.** Effect of NaCl (200 mM) on relative water content (RWC, %), stomatal conductance (gs, molm^−^^2^ s^−^^1^), transpiration rate (E, mmol H_2_O^−2^ S^−1^) and membrane stability index (MSI, %) in *A.gerrardii* Benth treated with and without 24-epibrassinolide. Data presented is mean of 10 replicates.

Treatments	Relative water content RWC (%)	Stomatal conductance(gs)	Transpiration rate(E)	Membrane stability index(MSI)
Control	84.5 ± 2.4b	0.32 ± 0.07b	5.96 ± 0.31b	85.9 ± 1.7b
EBL	90.6 ± 2.5a	0.52 ± 0.09a	6.92 ± 0.46a	94.4 ± 1.9a
NaCl	67.3 ± 1.9d	0.15 ± 0.02d	3.06 ± 0.25d	48.8 ± 1.1c
NaCl + EBL	76.4 ± 3.0c	0.26 ± 0.03c	5.08 ± 0.32bc	80.1 ± 1.5b
LSD at: 0.05	5.12	0.14	0.53	3.47

**Figure 1.**
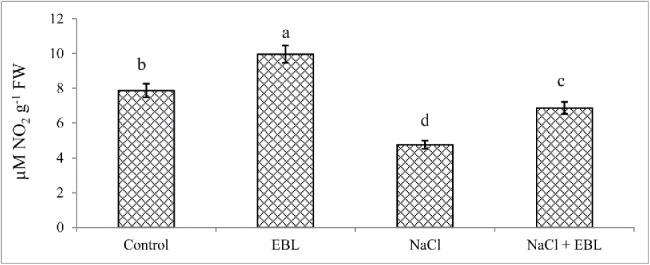
Effect of NaCl (200 mM) on nitrate reductase activity in *Acacia gerrardii* Benth treated with 24-epibrassinolide. Data present is mean of 10 replicates.

Plants treated with NaCl (200 mM) exhibited a 39.5% decline in nitrate reductase activity which was, however, recovered by 30.6% after the exogenous application of EBL. Applying EBL to control plants enhanced nitrate reductase activity by 21.2% (Fig. 1).

**Figure 2.**
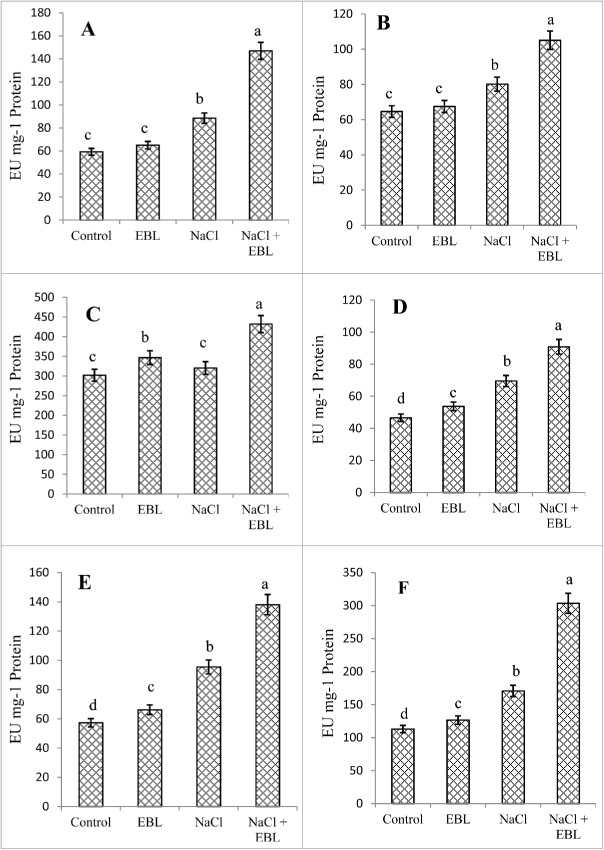
Effect of NaCl (200 mM) on superoxide dismutase (A), catalase (B), ascorbate peroxidase (C), glutathione reductase (D), dehydroascorbate reductase (E) and monodehydroascorbate reductase (F) activity in *Acacia gerrardii* Benth treated with 24-epibrassinolide. Data present is mean of 10 replicates.

Results depicting the effect of NaCl and exogenous EBL on the activity of antioxidants are shown in Figure 2A-F. NaCl resulted in a 32.9%, 19.3%, 6.0%, 33.0%, 40.0% and 33.9% increase in SOD, CT, APX, GR, DHAR and MDHAR activity, respectively while the application of EBL further increased the activity of these enzyme systems by 39.7%, 23.7%, 25.9%, 23.4%, 31.1%, and 43.9%, respectively more than NaCl^−^treated plants. However, EBL applied to control plants stimulated the activity of these enzyme systems by 8.8%, 4.2%, 12.9%, 13.2%, 13.5% and 10.8%, respectively more than control plants (Fig. 2A-F).

## Discussion

Plant growth regulators augment plant growth and performance in a range of environments, but the importance and the role of BRs in plant growth regulation has received the attention of researchers only in the last few years. BRs reportedly aid plants in counteracting the negative effects of different stresses. In the present study, the role of exogenously applied BR (24-EBL) in mitigating the deleterious effects of NaCl in *A. gerrardii* plants was evaluated. The application of 24-EBL resulted in a significant increase in growth and biomass accumulation. BRs regulate growth and developmental processes in plants by inducing cell proliferation and expansion by affecting the differentiation of vascular tissues.^32^ Excess uptake of NaCl obstructs the functioning of membrane transporters responsible for cellular expansion and alters the progression of the cell cycle leading to altered growth.^21^ A decline in growth due to salinity and its amelioration by the exogenous application of BR observed in our study confirm with the results of other researchers in *Cicer arietinum*,^33^ *Vigna radiata*^19^ and *Vigna sinensis*.^34^ In salt stressed *Cucumis sativa*, Fariduddin et al.^23^ demonstrated that the exogenous application of EBL significantly mitigated the NaCl^−^induced decline in growth and biomass accumulation. Plant steroidal hormones potentially control the negative effects of abiotic environmental stresses including high salinity, water, and metals.^21,35^ BRs also interact with other growth regulators to up-regulate signaling events, resulting in improved cell elongation and germination.^35^

In the present study, an NaCl^−^induced, negative impact on growth parameters was observed. This directly caused a significant decline in the synthesis of photosynthetic pigments, but these negative responses were improved by the exogenous application of EBL. The application of EBL has been shown to improve the content of photosynthetic pigments and associated photosynthetic attributes such as stomatal conductance, electron transport, PSII activity and carbonic anhydrase activity in *Cucumis sativus* under salt and/or copper stress.^36^ In this study, NaCl^−^induced salinity negatively affects pigment biosynthesis thereby declining photoassimilate production and growth, but EBL improved chlorophyll content and stomatal conductance, showing the protective role of EBL against NaCl^−^induced oxidative stress. The findings of Anuradha and Rao^37^ for *Oryza sativa* L., Ali et al.^33^ for *Cicer arietinum* and Hayat et al.^19^ for *Vigna radiata* reported in similar trend to our study that photosynthesis and antioxidant system decreased in the presence of salt stress, however the application of polyhydroxysteroids (brassinosteroids) alleviated the adverse impact of salinity. Yuan et al.^38^ demonstrated that EBL protects the structural integrity of chloroplasts by protecting grana from stress-induced disintegration. EBL promotes photosynthesis and improves water uptake leading to a significant enhancement of stomatal functioning, and leading to regulated transpiration. The enhancement of chlorophyll due to exogenous application of EBL in control and stressed plants in the present study may be due to the upregulation of chlorophyll-synthesising enzymes that improved the protection of photosynthetic pigments. Excess NaCl interferes with the structure and stability of the photosynthetic pigment-protein complex, and when coupled with the increase in chlorophyll degradation due to upregulated chlorophyllase activity, this leads to chlorophyll degradation.^39^ In the present study, exogenous application of EBL to *A. gerrardii* plants resulted in an enhancement of carotenoid contents, adding to the antioxidant function against NaCl^−^induced photodamage and protecting photosynthetic electron transport.^16^ Similar to this finding in rice, Anuradha and Rao^37^ noted improved carotenoid synthesis after the exogenous application of EBL. In the present study, EBL enhanced photosynthetic pigment content, possibly due to its impact on the uptake of ions such as magnesium, an important part of chlorophyll, which would otherwise be inhibited by excess NaCl. In the same context, Li et al.^40^ reported that, overexpression of brassinosteroid (BR) biosynthesis or signaling is a promising strategy improved crop yield as well as quality, photosynthetic capacity through activation of Calvin cycle enzymes in tomato.

From the results of the present study, it is evident that treatment of EBL reduced the over-accumulation of free radicals in *A. gerrardii* plants. Free radicals have the potential to diffuse through membrane aquaporins over larger distances leading to the hampered functioning of biologic membranes.^41^ The efficient elimination of ROS in EBL-supplemented plants induced by high antioxidant activity justifies the growth promotive role of EBL through its involvement in membrane protection from toxic ROS, thereby leading to increased membrane stability. The enhancement of ROS (antioxidant enzymes) in the present study may be due to reduced RWC which obstructed its diffusion from production sites while EBL protected *A. gerrardii* plants by maintaining RWC. Weisany et al.^9^ reported that, decline RWC due to salt stress resulted significant increase in lipid peroxidation accompanied with decrease photosynthetic capacity in *Glycine max*, however the alleviation of salt stress was by regulation and maintaining RWC and lipid peroxidation. Accordingly, Chen et al.,^42^ while studying the proteomic responses in rice, noted an increase in lipid peroxidation due to upregulation of lipoxygenase which resulted in the quick denaturation of membrane lipids. Formerly, Hayat et al.^19^ demonstrated that exogenous application of EBL significantly improved membrane stability by reducing ROS production and improving the antioxidant system for protecting the photosynthetic apparatus. The improved RWC in EBL-supplemented *A. gerrardii* plants may be due to the enhanced accumulation of osmolytes including free proline, sugars, and glycine βine.^19^ EBL-induced improvement in RWC may have contributed to the regulation of cell swelling and therefore to morphogenesis.

Nitrate reductase catalyzes the first rate-limiting step in nitrate assimilation. Improved nitrate reductase activity in EBL-treated *A. gerrardii* plants in this study was also reported in *Oryza sativa*^22^ and *Vigna radiata*^19^ treated with brassinosteroids to regulate the adverse impact of salt stress. In salt-stressed *Cucumis sativus*, Fariduddin et al.^36^ demonstrated that the application of 28-homobrassinolide mitigated the NaCl^−^induced decline in nitrate reductase activity. Improved nitrate reductase activity results in increased availability of nitrogen precursors for the enhanced synthesis of amino acids. In the present investigation, EBL-induced enhancement of nitrate reductase activity may have contributed to the improved synthesis of amino acids leading to better NaCl stress tolerance and the synthesis of certain phytohormones such as ethylene.^13^ *A. gerrardii* plants exposed to salinity and treated with or without EBL displayed a significant alteration in the expression of antioxidant enzymes. Together with our report in the present study, that EBL induces upregulation of apoplastic ROS in *Cicer arietinum*,^33^ *Vigna radiate*,^19^ *Cucumis sativus*^36^ and *Brassica juncea*^8^ under salt stress. This is supported by the observation that Chickpea plants exposed to NaCl treatments induced several isozymes of SOD, CT, APX and POD that mediated the protection against toxic ROS.^15^ SOD is the frontline enzyme component of the antioxidant system, and mediates the scavenging of superoxide radicals. Increased SOD activity due to the application of EBL may have benefitted NaCl^−^treated *A. gerrardii* plants by reducing the formation of Haber-Weis reaction substrates resulting in the limited formation of the toxic hydroxyl radical. Unlike EBL-treated plants, reduced SOD activity in NaCl^−^stressed plants may have exhibited an increase in the accumulation of radicals, thereby affecting membrane functioning. Our results showing an increase in the activity of antioxidant enzymes as a result of EBL application is supported by several other studies in *Brassica juncea*^43^; *Cucumis sativus*^23^ and *Vigna radiata*.^19^ Improved antioxidant metabolism leads to the speedy elimination of free ROS, thereby leading to the stability of cellular metabolism. Increased peroxidase activity enhances the synthesis of cell wall components by improving the formation of lignin and protects plants from biotic infestation.^14^ CT and APX share a common substrate, H_2_O_2_, and play an ancillary role against stress, and their higher activity improves salinity tolerance.^8,15,19^ In salinity-stressed *Brassica juncea*, Sarwat et al.^8^ demonstrated the significant enhancement of CT and APX activity, which protected membranes and photosynthesis from oxidative damage. The exposure of plants to NaCl stress improves the expression level of antioxidant enzymes.^44^ Besides SOD and CT, enzymes including APX, MDHAR, DHAR and GR are among the most versatile players in the elimination of ROS. APX, MDHAR, DHAR and GR are induced as key enzymatic components of the efficient ROS-scavenging pathway, the ascorbate-glutathione cycle.^16^ Ahmad et al.^11^ demonstrated that upregulation of the enzymes of the ascorbate-glutathione cycle increased the tolerance of *B. juncea* to salt stress. This cycle involves a series of redox reactions catalyzed by APX, MDHAR, DHAR and GR that scavenge H_2_O_2_ in the chloroplast, mitochondria, and cytosol. Similar to our findings, Ahmad et al.^11^ and Hashem et al.^7^ also noticed an increase in APX and GR activity in *B. juncea* and *Ocimum basilicum* after NaCl treatment. In the present study, application of EBL further enhanced the activity of the antioxidant system in *A. gerrardii* plants. The optimal activity of APX and GR in the ascorbate-glutathione cycle depends on the efficient working of MDHAR and DHAR.^11^ In the present study, EBL-induced upregulation of the activity of APX, MDHAR, DHAR and GR prevented photo-oxidative damage in *A. gerrardii* plants by maintaining the levels of NADP so that photosynthetic electron transport was weakly affected by NaCl. Li et al.^40^ demonstrated that tomato plants (*Solanum lycopersicum*) exhibiting upregulation of BR synthesis showed the enhanced synthesis of GSH and AsA, leading to the activation of MDHAR and DHAR and resulting in the optimal supply of AsA and GSSG to APX and GR, respectively. In the present study, the improved activities of enzymes of the ascorbate-glutathione pathway following the application of EBL may have also benefited *A. gerrardii* plants by enhancing the synthesis of AsA and GSH. Improved MDHAR and DHAR activity due to the application of EBL has been reported in tomato.^40^ In addition to this improvement in the activities of APX, MDHAR, DHAR and GR, EBL-treated plants may have maintained a higher GSH/GSSG ratio, thus protecting redox-dependent metabolic processes.^19^ Glutathione-dependent antioxidative defense and redox reactions play a key role in stress mitigation. In the present study, EBL-mediated enhancement of the activities of ascorbate-glutathione pathway enzymes justifies the importance of EBL as a suitable candidate stress marker.^45^

## Conclusion

In conclusion, exogenously applied EBL significantly mitigated the negative effects of NaCl in *A. gerrardii* plants by up-regulating the antioxidant defense system. NaCl reduced growth by impeding photosynthesis, nitrate reductase and RWC. However, EBL proved to be beneficial, and protected vital functions of *A. gerrardii* plants under NaCl stress. Membrane protection and enhanced chlorophyll synthesis in EBL-treated plants justifies the ameliorative role of EBL against NaCl stress in *A. gerrardii*.
